# Supplementary material for: Occupational inequalities in the prevalence of COVID-19: A longitudinal observational study of England, August 2020 to January 2021
Source: PLoS One. 2023 Apr 5;18(4):e0283119. doi: 10.1371/journal.pone.0283119 (PMC10075431; doi:10.1371/journal.pone.0283119)
Supplement: S1 File — (PDF) [file pone.0283119.s001.pdf]

Appendix

Table A: Descriptions of work sector categories.

Work Sector	Example occupations
ICT	IT technician, software engineer, programmer, web designer
Education	Teacher, lecturer, school workers
Health care	Doctor, nurse, dentists, health professionals, pharmacists
Social care	Carer, social workers, welfare professionals
Transport	Bus driver, logistics, storage firms, taxi driver
Retail sector	Shop assistant, retail cashier, check-out operators
Hospitality	Server, waiting staff, bar staff, chef
Food production	Farmer, agricultural labourer, butcher, baker
Personal services	Hairdresser, barber, cleaner, beautician
Finance	Insurance, banking, accountant,
Manufacturing	Construction, skilled and non-skilled trades, engineers
Civil service	Local Government worker, health and safety officer,
Armed forces	Solider, naval officer, air forces pilot
Entertainment	Actor, artist, musician, recreational officer

Table B: Sample summary statistics.

Variable	Mean (SD)
Age	53.96 (17.1)
**Variable**	**Percentage (%)**
Test outcome	
Negative	96.9
Positive	0.9
Void	2.2
Sex	
Male	46.6
Female	53.4
Ethnicity	
White British	87.9
Any other ethnic group	2.0
Any other white background	4.1
Chinese	0.5
Indian	1.9
Pakistani	0.4
Black-African	0.4
Black Afro-Caribbean	0.3
Mixed-White & Asian	0.4
Mixed-White & Black	0.4
White-Irish	1.7

Work status	
Employed	47.1
Self-employed	4.3
Furloughed	3.9
Not working	42.6
Student	2.1
Work sector	
ICT	7.6
Education	14.4
Health care	11.4
Social care	3.0
Transport	4.1
Retail sector	7.5
Hospitality	3.3
Food production	1.7
Personal services	1.4
Finance	8.7
Manufacturing	10.0
Civil service	7.1
Armed forces	0.4
Entertainment	2.9
Other	16.5
Month	
August	6.0
September	12.9
October	23.1
November	20.5
December	18.3
January	19.2

Table C: Percentage of records with missing data.

Variable	Missing (%)
Test outcome	3.57
Age	0.00
Sex	0.00
Ethnicity	0.02
Work status	0.06
Work sector	3.57
Month	0.00
Geography	10.98
Travel abroad	3.20
Household size	0.00

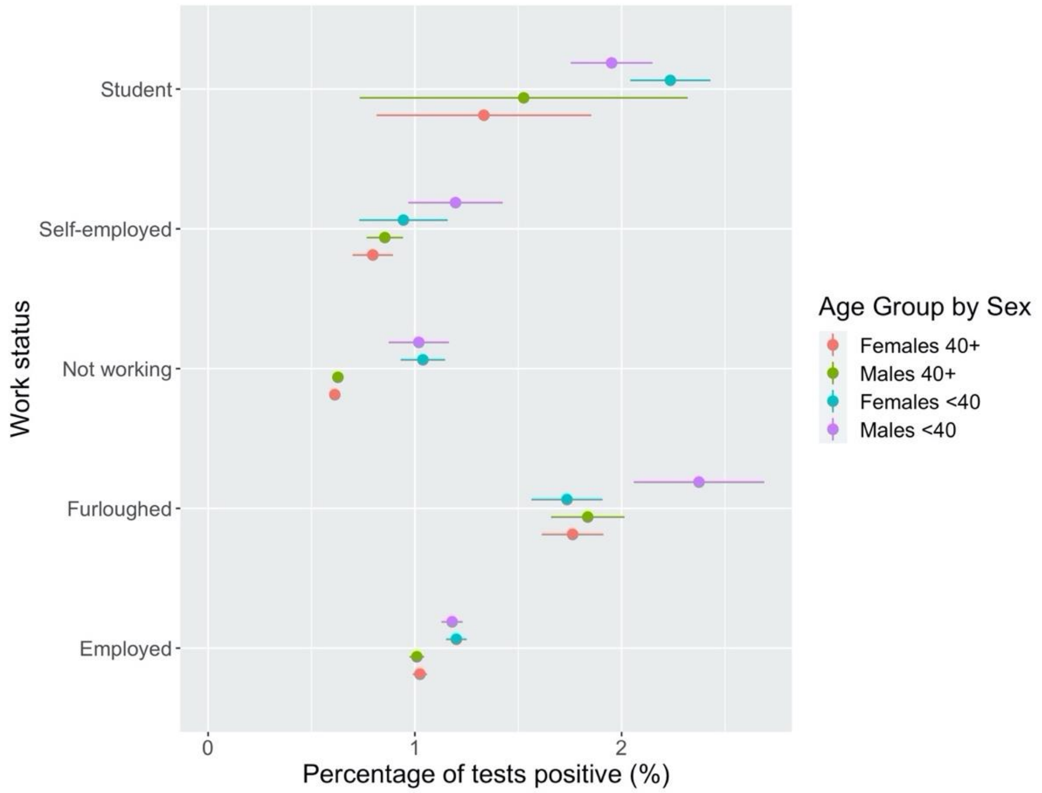

Figure A: COVID-19 Prevalence by work status and sex for individuals by age group.

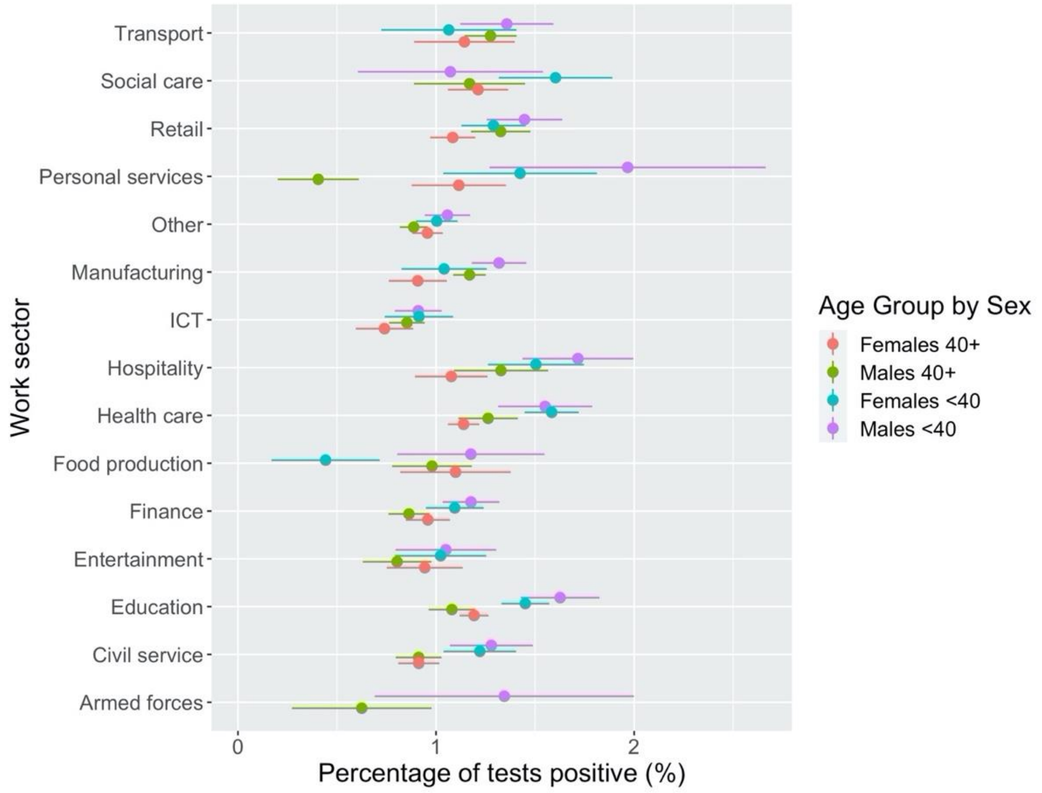

Figure B: COVID-19 prevalence by work sector and sex for individuals by age group. Note: estimate for females employed in the armed forces excluded due to counts <10 to preserve ONS data disclosure standards.

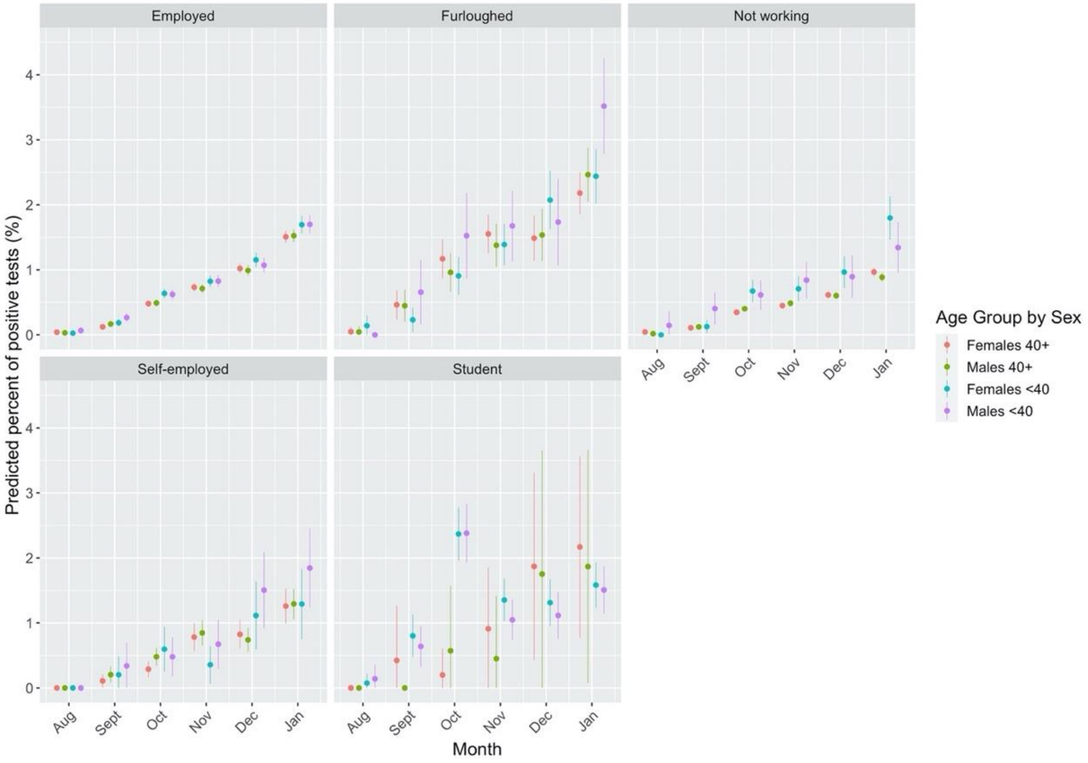

Figure C: Predicted probability of testing positive for COVID-19 by work status, sex and month stratified by age group.

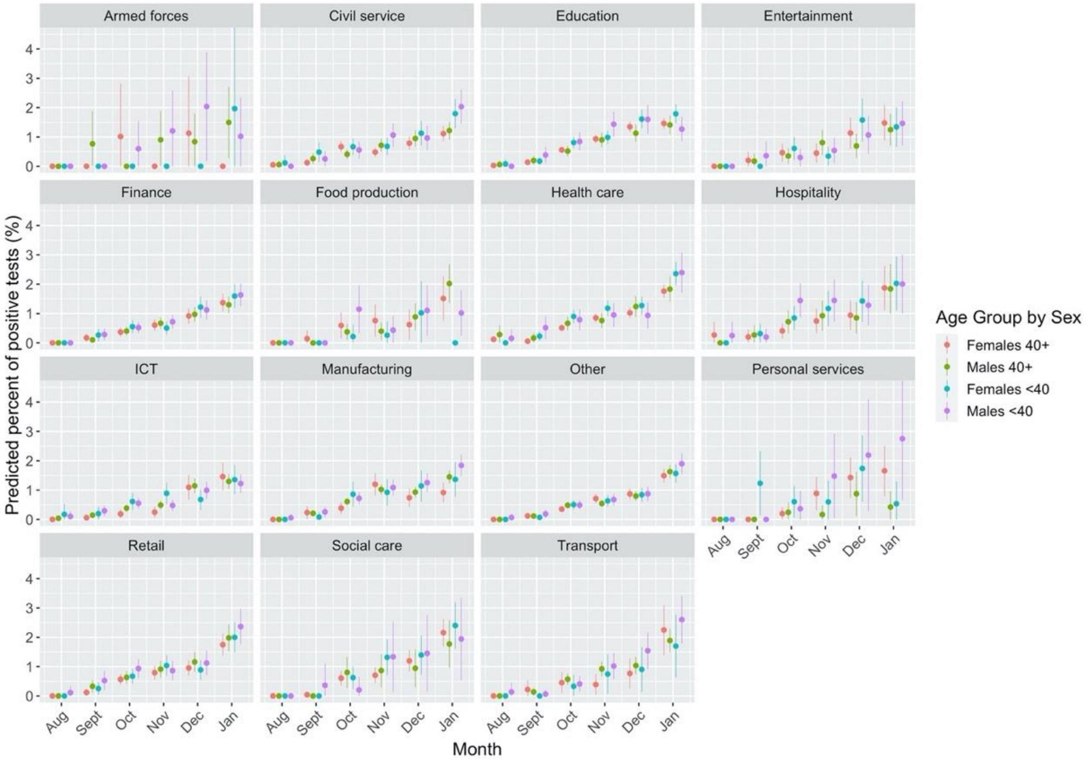

Figure D: Predicted pry of testing positive for COVID-19 by work sector, sex and month stratified by age group.
