# Supplementary material for: Occupational inequalities in the prevalence of COVID-19: A longitudinal observational study of England, August 2020 to January 2021
Source: PLoS One. 2023 Apr 5;18(4):e0283119. doi: 10.1371/journal.pone.0283119 (PMC10075431; doi:10.1371/journal.pone.0283119)
Supplement: Attachment — Submitted filename: PONE-D-21-18992.docx [file pone.0283119.s002.docx]

Overall results and discussion needs some more work. However, introduction and methodology need to be cleared.

The aim of this study is to evaluate how occupational inequalities in the prevalence of COVID-19 vary 59 across England and their possible explanatory factors, but there is almost nothing in the introduction section.

Moreover, how it is possible to develop interventions to minimize transmission risk for managing COVID-19 and 63 future pandemics by using the secondary data with limited variables and when selection bias is one of the biggest limitation.

I think it’s the data from the surveillance and not the primary data, as it was not collected for the research to develop interventions.

They need to change their outcome of this paper and its significance.

The model development information is missing; I could see univariate analysis only.

Please take of typo errors

In discussion authors never shared what intervention they are suggesting.

Discussion need to more structured and need to explain their results more clearly.
