# Supplementary material for: Occupational inequalities in the prevalence of COVID-19: A longitudinal observational study of England, August 2020 to January 2021
Source: PLoS One. 2023 Apr 5;18(4):e0283119. doi: 10.1371/journal.pone.0283119 (PMC10075431; doi:10.1371/journal.pone.0283119)
Supplement: Attachment — Submitted filename: Response to reviewers.docx [file pone.0283119.s003.docx]

**Response to reviewers**

**Comment from authors:** We would like to thank both the Editor and the Reviewer for their helpful comments and the opportunity to revise our paper further. We have worked hard on revising our paper in line with the suggestions which have strengthened the narrative and quality of the paper. If there are further changes please let us know. Below, we provide a point by point response to each comment (starting first with the Editorial comments, then the reviewer comments). Where page numbers are provided, we refer to the tracked changes version of the manuscript. Thank you again.

**Journal Requirement Revisions:**

**Journal comment:** Please ensure that your manuscript meets PLOS ONE's style requirements, including those for file naming. The PLOS ONE style templates can be found at 
<https://journals.plos.org/plosone/s/file?id=wjVg/PLOSOne_formatting_sample_main_body.pdf> and 
<https://journals.plos.org/plosone/s/file?id=ba62/PLOSOne_formatting_sample_title_authors_affiliations.pdf>

**Response:** We have updated the manuscript to match the style requirements.

**Journal comment:** Thank you for stating the following financial disclosure: "This work was supported by the Economic and Social Research Council [grant number ES/L011840/1]."
 
Please state what role the funders took in the study.  If the funders had no role, please state: "The funders had no role in study design, data collection and analysis, decision to publish, or preparation of the manuscript." 
If this statement is not correct you must amend it as needed. 
Please include this amended Role of Funder statement in your cover letter; we will change the online submission form on your behalf.

**Response:** The funders had no role in study design, data collection and analysis, decision to publish, or preparation of the manuscript. We have mentioned this in the cover letter to make this clear now. The PLOS One team asked us to remove this information from the acknowledgements from the revision, which we have to confirm to their request so we hope that mention here is suitable for the revision.

**Journal comment:** Thank you for stating the following in your Competing Interests section:  "None declared" 
Please complete your Competing Interests on the online submission form to state any Competing Interests. If you have no competing interests, please state "The authors have declared that no competing interests exist.", as detailed online in our guide for authors at <http://journals.plos.org/plosone/s/submit-now> 
 This information should be included in your cover letter; we will change the online submission form on your behalf.

**Response:** The authors have declared that no competing interests exist. What have updated this on the system. We have updated this on the paper now.

**Journal comment:** In your Data Availability statement, you have not specified where the minimal data set underlying the results described in your manuscript can be found. PLOS defines a study's minimal data set as the underlying data used to reach the conclusions drawn in the manuscript and any additional data required to replicate the reported study findings in their entirety. All PLOS journals require that the minimal data set be made fully available. For more information about our data policy, please see <http://journals.plos.org/plosone/s/data-availability>.

Upon re-submitting your revised manuscript, please upload your study’s minimal underlying data set as either Supporting Information files or to a stable, public repository and include the relevant URLs, DOIs, or accession numbers within your revised cover letter. For a list of acceptable repositories, please see <http://journals.plos.org/plosone/s/data-availability#loc-recommended-repositories>. Any potentially identifying patient information must be fully anonymized.

Important: If there are ethical or legal restrictions to sharing your data publicly, please explain these restrictions in detail. Please see our guidelines for more information on what we consider unacceptable restrictions to publicly sharing data: <http://journals.plos.org/plosone/s/data-availability#loc-unacceptable-data-access-restrictions>. Note that it is not acceptable for the authors to be the sole named individuals responsible for ensuring data access.

We will update your Data Availability statement to reflect the information you provide in your cover letter.

**Response:** We have added more information so that data access is clear (this has been updated on the online system, as well as in the cover letter):

The ONS’s COVID-19 Infection Survey (CIS) is an individual level survey that collects population level data about people who live in the UK to estimate the prevalence of COVID-19. They collect a range of data including demographic and social data, as well as test everyone for COVID-19. These data contain sensitive and personal information about individuals (including information about date of birth and health information) . Such data cannot be openly shared to ensure privacy and meet statistical disclosure laws (i.e., researchers cannot legally distribute).

Access requires approval by the ONS who act as the data owner and do not allow researchers to hold or distribute their data. Data are free to access for accredited researchers within the ONS’s Secure Research Service (SRS). The SRS is a trusted researcher environment which provides free access to the ONS’s sensitive data (it acts as a secure data repository). More information about the process, including how to apply to access the data, can be found here: <https://www.ons.gov.uk/aboutus/whatwedo/statistics/requestingstatistics/secureresearchservice>

All analytical code used to process the data and replicate the results in the paper can be found here: <https://github.com/markagreen/occupational_inequalities_CIS>

Metadata: <https://ons.metadata.works/browser/dataset?id=293>

**Journal comment:** We note that you have stated that you will provide repository information for your data at acceptance. Should your manuscript be accepted for publication, we will hold it until you provide the relevant accession numbers or DOIs necessary to access your data. If you wish to make changes to your Data Availability statement, please describe these changes in your cover letter and we will update your Data Availability statement to reflect the information you provide.

**Response:** This has been responded to and revised in the above/previous comment.

**Journal comment:** Please include captions for your Supporting Information files at the end of your manuscript, and update any in-text citations to match accordingly. Please see our Supporting Information guidelines for more information: <http://journals.plos.org/plosone/s/supporting-information>.

**Response:** These have now been added to the bottom of the manuscript (p26 tracked version) and we have updated citations within text accordingly.

**Reviewer comments**

**Reviewer #1:** Overall results and discussion needs some more work. However, introduction and methodology need to be cleared.

**Reviewer comment:** The aim of this study is to evaluate how occupational inequalities in the prevalence of COVID-19 vary across England and their possible explanatory factors, but there is almost nothing in the introduction section.

**Response:** We have now expanded the introduction to bring greater depth of the context of our study. We have emphasised making it clearer to the reader why this piece of work was important, what is novel and why it matters. We have also made reference to the importance of this work for informing intervention development, in line with other comments during your review. We have opted for a length similar to other PLoS One articles (i.e., try to be concise where possible). We have also changed the structure from three paragraphs, to 5 paragraphs so that it flows better and guides the reader through the importance of the problem we are investigating. This also helps accommodate the additional material.

Specifically, additions include more detail on why it is important to look at occupational differences:

“*Most of this existing research has focused on severe outcomes or a narrow range of occupations/work sectors, meaning that we have less evidence of how SARS-CoV-2 infections vary across occupations or work settings. Preventing infections and exposure to SARS-CoV-2 will help to reduce severe COVID-19 outcomes. Understanding which occupations had higher or lower infections is imperative for designing preventative work-place interventions for managing COVID-19 and preparing for future pandemics*.” (pp3-4 tracked version)

Additional text has been added on why occupation is important to consider if we want to understand inequalities:

“*Occupational status intersects with age, sex, ethnicity and deprivation. For example, individuals from deprived neighbourhoods or Black and Asian ethnic groups are more likely to be employed in occupations that were unable to work from home or in ‘essential’ front facing roles (3,15); the same groups who have seen higher hospitalisations and mortality related to COVID-19. Occupation type is therefore a fundamental driver of exposure to SARS-CoV-2 in employed populations, meaning that occupation may partly explain or amplify health inequalities relating to COVID-19 (5,16).*” (p4 tracked version)

We have also made some minor changes the wording of the introduction either to clarify statements (e.g., line 58 tracked version) or to state the importance for intervention development (lines 77-80 tracked version).

We hope that this additional context, appended to the original introduction, brings it more in line with the expectations for an introduction.

**Reviewer comment:** Moreover, how it is possible to develop interventions to minimize transmission risk for managing COVID-19 and future pandemics by using the secondary data with limited variables and when selection bias is one of the biggest limitation. I think it’s the data from the surveillance and not the primary data, as it was not collected for the research to develop interventions.

**Response:**

The use of secondary data in epidemiology/public health is a common practice in studying population health issues (like our research question). It is a valid approach that has been used widely and PLoS One publishes a lot of papers that are based on secondary data analysis. Using secondary data here brings a lot of benefits to conducting our research project that would have not been possible if we had gone down a primary data route (especially in collecting a large data set as we needed). To help make this clearer in our paper, we have added a paragraph detailing this so that it is clear to the reader. The paragraph reads:

“*The ONS Covid Infection Survey (CIS) was used as our data source. The CIS is a representative random sample survey of the population in England used to monitor trends in COVID-19 (18). While primarily used as a surveillance tool for COVID-19, CIS was also designed so that the data could be re-used by researchers as a secondary data resource for understanding population health issues relating to the pandemic. Secondary data are useful resources since they allow for efficient collection of large and complex data that saves time and costs over primary data collection (19). This is valuable for our study since (i) the rarity of our outcome (0.9% in this study) means we require a large sample size to find robust associations, and (ii) the CIS was designed to collect a national-level representative sample that minimises selection bias meaning our findings can be generalisable*.” (p5 tracked version)

Selection bias, as described in the above paragraph, is not a big issue here since the secondary data source we used was a large national level survey that is representative and based on random sampling. This helps our results be generalisable and relevant; the representative design of the survey helps minimise issues of selection bias and is a strength of using the data source.

We also note later that since all individuals are swabbed and tested for SARS-CoV-2, we minimise selection bias why relying on self-reported testing data. We have added a statement to make this clear now:

“This helps to minimise issues in secondary data based on self-reported testing records due to selection bias in who tests and who registers tests (18).” (lines 105-106 tracked version)

Secondary data has value to population health researchers beyond evaluating a single intervention. In our case, it allows us to understand the extent of occupational inequalities in COVID-19 risk and the reasons behind this. These issues are new and we lack detailed evidence on the reasons behind why certain occupational groups may be a higher/lower risk; important for learning from the pandemic. We can then use this information to try and design relevant interventions – our study is one piece of evidence in this process (this is no different to how social epidemiology and public health research operates too). We discuss specific additional changes and content about interventions later in the responses (see below/discussion section).

**Reviewer comment:** They need to change their outcome of this paper and its significance.

**Response:** The aim of our paper was “to evaluate how occupational inequalities in the prevalence of COVID-19 vary across England and their possible explanatory factors”. Our outcome variable is ‘whether an individual had a positive SARS-CoV-2 test or not’ which is the primary and established outcome measure for understanding prevalence of COVID-19 (i.e., a positive test is required to see who has COVID-19). We therefore believe that the correct outcome was used.

**Reviewer comment:** The model development information is missing; I could see univariate analysis only.

**Response:** Our analyses included multivariable multi-level models (i.e., adjustment for all explanatory variables in the model). Please see Tables 1 and 2 which detail the results from the fully adjusted models here. Figures 6 and 7 were also generated from using fully adjusted models as well. To avoid confusion here to the reader, we have made it explicit in the statistical analysis section that we used multivariable models now (p7).

We have also added a further text to clarify our model development so that it is clear what we did. It now reads: “*Multi-level binomial regression models were used to analyse the risk of COVID-19. Two models were used; one for employment status and one for occupational sector (our key exposure variables). Multivariable models were fully adjusted using a series of fixed effect variable representing our explanatory individual-level covariates that may explain differences in COVID-19 risk. Numeric values were z-score standardised to minimise issues with their different scales (age and household size). Two random effects are included: (i) participant ID (varying intercept) to account for repeat observations within the survey over time, and (ii) geographical area (varying intercept) to account for geographical inequalities in COVID-19 prevalence and therefore risk. Our model building process began through examining our key exposures and the random effects independently, prior to including all variables in the model, and then checking model assumptions. We present only the fully adjusted analyses here*.” (p8 tracked version)

We have also mentioned why we calculated the conditional mean values from our multivariable models so it was clearer to the reader: “*We estimate the conditional mean for each geographical area from our fully adjusted multivariable models. Through mapping these estimates, we can visually examine if there were distinct geographical patterns in COVID-19 during the study period to understand the role that location plays in our model and to identify the need for spatially targeted interventions*.” (p8 tracked version)

Finally, we have moved the content about the interaction effect models for estimating changes over time that was originally present in the results into the statistical analysis section of the methodology. This improves the structure of the paper through having all the methodological steps in one place. We hope that all of these changes have help make things clearer now.

**Reviewer comment:** Please take of typo errors

**Response:** We have gone through the paper carefully and make changes to address spelling and grammar issues. We do not detail all of the changes here for brevity, but they can be found on the tracked changes version of the paper.

**Reviewer comment:** In discussion authors never shared what intervention they are suggesting.

**Response:**

Our study is a piece of descriptive epidemiology (observational study) rather than evaluating an intervention. We have a large range of results/key findings, meaning there are quite different ways to recommend interventions based on them. We agree with you that we need to make it clearer the implications of our work (including possible interventions). To make it clearer what interventions might be needed based on our study (since our findings speak to a range of issues and potential interventions arising from), we have expanded our discussion section to make this clearer to the reader. Specifically, when talking about furloughed populations we have added:

“*Our findings would recommend careful educational messaging where future furlough schemes are introduced to help guide individuals who were furloughed in navigating their new risks, or encouraging employers to find ways to help individuals work from home rather than being furloughed where possible*.” (p18 tracked version)

When talking about issues with student populations we have added:

“*Improved ventilation and use of marks in indoor classroom settings during periods of high community prevalence may be needed to avoid similar patterns during term time (25). Such interventions may only be effective if paired with strategies around social experiences of University students*.” (p19 tracked version)

We also note that we have a long discussion about the importance of workplace interventions. It would be beyond the scope of our article to provide bespoke interventions for all occupational groups, hence why we provide a more general discussion.

“*Our findings suggest the need for better workplace interventions across diverse roles that can help contain COVID-19 transmission, whilst allowing individuals and employers to continue their social and economic activities (7). Occupational roles will need to further adapt to protect their employees from COVID-19. Minimising social contacts or mixing within occupational roles through sufficient preventative measures may be valuable. One study suggested that limiting the number of social contacts at work was the most important strategy for lowering the ‘R’ number if society keeps schools open (17). Repeat testing of employees may help to manage outbreaks, however testing behaviours can also widen inequalities (20). Support for lost earnings if individuals have to self-isolate will be key, especially as some of the work sectors identified here with higher prevalence (e.g., retail or hospitality) are characterised by low wages (16). The UK Government should also consider targeting particular work sectors considered at ‘higher risk’ of infections and introducing interventions in those areas when required. However, our findings of high prevalence of COVID-19 for furloughed and student populations demonstrates the need for broader strategies than just occupation-related interventions to help manage COVID-19 and tackle the drivers of health inequalities*.” (pp19-20 on tracked version)

We have also stated in our key results paragraph that this work matters for intervention design: “with work status and occupational group being one route for interventions aimed at tackling inequalities” (p18 tracked version).

Our paper now hopefully makes it clear potential strategies or areas to improve in terms of interventions/policy development.

**Reviewer comment:** Discussion need to more structured and need to explain their results more clearly.

**Response:** We have gone through our discussion again and revised the content to try and improve the clarity of the text. Our structure follows the STROBE observational checklist – so first paragraph describe key results, then provide the interpretation for the key results (explain in detail each one separately), then describe limitations of your work, then conclusion.
